# Spin–orbit interactions in plasmonic crystals probed by site-selective cathodoluminescence spectroscopy

**DOI:** 10.1515/nanoph-2023-0065

**Published:** 2023-04-05

**Authors:** Masoud Taleb, Mohsen Samadi, Fatemeh Davoodi, Maximilian Black, Janek Buhl, Hannes Lüder, Martina Gerken, Nahid Talebi

**Affiliations:** Institute of Experimental and Applied Physics, Kiel University, 24098 Kiel, Germany; Integrated Systems and Photonics, Faculty of Engineering, Kiel University, 24143 Kiel, Germany; Kiel, Nano, Surface, and Interface Science, Kiel University, 24098 Kiel, Germany

**Keywords:** angular momentum, cathodoluminescence, plasmonic crystal, spin-orbit coupling

## Abstract

The study of spin–orbit coupling (SOC) of light is crucial to explore the light–matter interactions in sub-wavelength structures. By designing a plasmonic lattice with chiral configuration that provides parallel angular momentum and spin components, one can trigger the strength of the SOC phenomena in photonic or plasmonic crystals. Herein, we explore the SOC in a plasmonic crystal, both theoretically and experimentally. Cathodoluminescence (CL) spectroscopy combined with the numerically calculated photonic band structure reveals an energy band splitting that is ascribed to the peculiar spin–orbit interaction of light in the proposed plasmonic crystal. Moreover, we exploit angle-resolved CL and dark-field polarimetry to demonstrate circular-polarization-dependent scattering of surface plasmon waves interacting with the plasmonic crystal. This further confirms that the scattering direction of a given polarization is determined by the transverse spin angular momentum inherently carried by the SP wave, which is in turn locked to the direction of SP propagation. We further propose an interaction Hamiltonian based on axion electrodynamics that underpins the degeneracy breaking of the surface plasmons due to the spin–orbit interaction of light. Our study gives insight into the design of novel plasmonic devices with polarization-dependent directionality of the Bloch plasmons. We expect spin–orbit interactions in plasmonics will find much more scientific interests and potential applications with the continuous development of nanofabrication methodologies and uncovering new aspects of spin–orbit interactions.

## 1 Introduction

**Figure 1:**
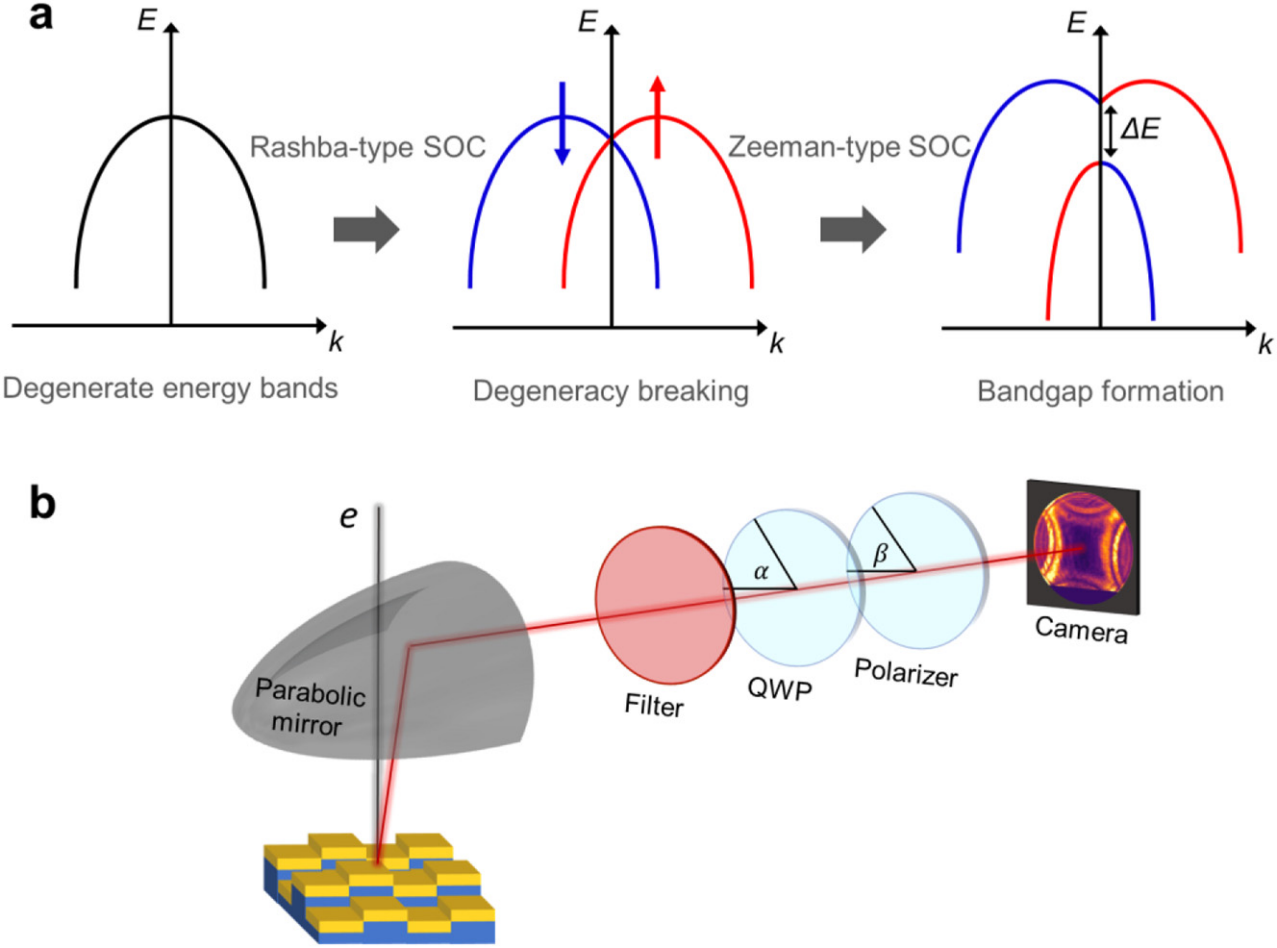
Spin–orbit interactions leading to energy splitting. (a) Degeneracy breaking and band splitting due to the Rashba-type and Zeeman-type spin–orbit coupling. (b) Experimental setup for angle-resolved CL polarimetry equipped with a filter, a quarter wave plate (QWP) and a linear polarizer to measure the polarization state of the CL emission of the sample. The sample is excited by an electron beam and the resulting CL emission is directed to a camera by a parabolic mirror (projected on the CCD camera is the *S*_0_ Stokes parameter of the lattice at *λ*_0_ = 800 nm [*E* = 1.55 eV]).

Spin–orbit interactions have raised a great deal of interest among the condensed-matter physics and material engineering communities owing to their potential applications for the design of the topological states of matter [1, 2] in the developing field of spintronics [3]. The main goal for this area of research is to generate and control spin-polarized states by manipulation of inversion and time-reversal symmetries in solids. The Rashba [4, 5] and Dresselhaus [6] effects serve as examples of the spin–orbit coupling (SOC) phenomena occurring when the inversion symmetry of a crystal is broken, for example at the interface between two materials. Even in the absence of an external magnetic field, the SOC effects induce an effective magnetic field leading to peculiar angular momentum distributions within the crystal. In general, these effects show up as additional SOC terms in the Hamiltonian of the system splitting the otherwise spin-degenerate energy bands by shifting the band structures of the electrons with opposite spins to opposite directions in momentum space (Figure 1a). Rashba effect is induced by breaking the symmetry of the crystal along the direction normal to an interface. The potential gradient along this direction induces an effective electric field that in the frame of the moving electron, is observed as a magnetic field, which couples to its spin. The optical counterpart of the Rashba-type SOC was presented in a series of studies [7–10] wherein right (left)-handed circular polarization of light plays the role of the spin-up (down) state. It was demonstrated that by bringing cavity photonic modes of different parities in resonant condition, via utilizing anisotropic materials such as liquid crystals, the photonic system emulates an effective spin–orbit interaction Hamiltonian underpinning the Rashba-Dresselhaus effect [11]. Moreover, various forms of metamaterials have been used as well by other groups to synthesize the effective required anisotropic behavior [7–10]. The exotic features of light, including spin–orbit interactions, can be used to manipulate the charge density wave of the two-dimensional electron gas excited inside heterostructures. These effects have important implications for the development of electronic and optoelectronic devices [12].

While the photonic Rashba effect displaces the oppositely polarized bands along the momentum axis, there exists a second SOC effect, i.e. the Zeeman effect, which shifts the bands along the energy axis. In contrast to the conventional Zeeman effect being necessarily induced by an external magnetic field [13], non-magnetic Zeeman-type splitting is observed in 2D transition-metal dichalcogenides (TMDCs) [14, 15] and 3D materials with specific symmetry point groups [16, 17]. Simultaneous existence of the Rashba-type and Zeeman-type effects in photonic systems modifies the angular momentum distribution in such a manner that induces a bandgap in the energy-momentum dispersion diagram (Figure 1a). This can be realized through proper arrangement of the building blocks of a plasmonic crystal, as we demonstrate in this paper.

Another manifestation of SOC in condensed-matter systems is the quantum spin Hall effect (QSHE) that gives rise to counter-propagating edge states with opposite spin orientations [18–20]. These topologically-protected edge states lie inside the bulk energy gap of inverted band-gap semiconductors, e.g. HgTe, leading to a quantized spin Hall conductance. The edge states associated with the QSHE sustain a spin-momentum locking feature, i.e. the states with different spins have different momenta and hence different propagation directions. QSHE of light implies that guided or surface electromagnetic waves with opposite transverse spin angular momenta (SAM) propagate in opposite in-plane directions [21–25]. Upon interacting with an arbitrary scatterer, a surface wave of a given circular polarization is scattered along a certain direction determined by the state of polarization and the direction of the surface wave impinging at the scatterer. The QSHE of light provides a unique framework for interpreting optical wave phenomena that carries topological and quantum behavior, facilitating SAM-to-direction coupling and unidirectional excitation of guided or surface modes in plasmonic crystals and metasurfaces.

In analogy to the spin–orbit interactions in molecules, one can write the SOC Hamiltonian of a photonic system in the form of *A*_SO_**L** ⋅ **S** where **L** and **S** denote the orbital and spin components of the total angular momentum of light **J** and *A*_SO_ is the spin–orbit parameter [26]. Therefore, we relate the band splitting Δ*E* (shown in Figure 1a) to the scalar product of the spin and orbital angular momenta of light. In general, the energy of a trivial system is quadratic with respect to the electromagnetic fields and can be written as:

(1) $$W=\frac{1}{2}\left(E\cdot D^{*}+B\cdot H^{*}\right)=\frac{1}{2}\varepsilon E^{2}+\frac{1}{2\mu }H^{2}$$

and the Hamiltonian is thus defined as:

(2) $$\hat{H}=\frac{1}{2}\varepsilon E^{2}+\frac{1}{2\mu }H^{2}$$

For topological systems with a magnetoelectric effect, Wilczek [27] showed that the energy expression includes an extraordinary *θ***E** ⋅ **B** term in addition to the ordinary quadratic terms, where *θ* is a constant parameter related to the magnetoelectric polarizability. For a nonzero value of *θ*, the electric displacement **D** and the magnetic field **H** are functions of the magnetic induction **B** and the electric field **E** respectively, and can be written as:

(3) $$D=\varepsilon E-\frac{\theta \alpha }{\pi }B$$

(4) $$H=\frac{B}{\mu }+\frac{\theta \alpha }{\pi }E$$

where *α* is the fine structure constant. In this case, the behavior of the system is described by the modified Maxwell’s equations considering the magnetoelectric effects, called Axion electrodynamics [27, 28].

The proposed interaction Hamiltonian based on axion electrodynamics signifies a theoretical framework for understanding the behavior of surface plasmons in materials with strong spin–orbit coupling. This model offers insights into how light can interact with such materials and breaks the degeneracy of surface plasmons, which is an important phenomenon in the field of plasmonics. The model predicts that the spin–orbit interaction of light can induce an effective magnetic field in the material, which can in turn affect the behavior of surface plasmons. This insight could be useful in the design and engineering of plasmonic metamaterials that effectively show axionic behaviors and provides a new roadmap to manipulate the properties of surface plasmons.

In this paper, we propose a plasmonic crystal composed of a multilayer SiO_2_/ITO/Au configuration with a unit cell that exhibits varying ITO layer heights (Figure 1b). We argue that the SOC effects in our plasmonic crystal induce peculiar spin and orbital angular momentum distributions giving rise to extraordinary terms in the form of **E** ⋅ H* in the Hamiltonian. Thus, our crystal behaves in an effective medium approach, as a medium for hosting Axion electrodynamics. This effect comes into view as degeneracy breaking of plasmonic modes and formation of a bandgap in the energy-momentum dispersion diagram and splitting of circular patterns in the angle-resolved Stokes parameters obtained from CL experiments.

For a detailed study of the SOC effects in our crystal, we excite the sample with subwavelength control of the electron impact position in a CL polarimetry setup and analyze the polarization state of the angle-resolved CL patterns. For this purpose, we measure the intensity of the emitted light from the sample in six different polarization states (0°, 90°, 45°, and −45°, right- and left-handed circular polarizations) and calculate the Stokes parameters as:

$$S_{0}=I_{0{}^{\circ}}+I_{90{}^{\circ}}$$

(5) $$S_{1}=\left(I_{0{}^{\circ}}-I_{90{}^{\circ}}\right)/S_{0}$$

$$S_{2}=\left(I_{45{}^{\circ}}-I_{-45{}^{\circ}}\right)/S_{0}$$

$$S_{3}=\left(I_{\text{RCP}}-I_{\text{LCP}}\right)/S_{0}$$

where *I*_0°_, *I*_90°_, *I*_45°_ and *I*_−45°_ denote the intensities of the emitted light polarized in azimuthal angles *φ* = 0°, 90°, 45°, and −45° with respect to the *x*-axis or *φ* = 0°, *I*_RCP_ and *I*_LCP_ are the intensities of the right- and left-handed circularly polarized light. The parameter *S*_0_ describes the total intensity of the emitted light and the other three parameters, showing the degree of linear, diagonal and circular polarizations, respectively, are normalized to the value of *S*_0_. One can depict the Stokes parameters as a function of azimuthal (*φ*) and polar angles (*θ*) to show the angular distribution of the light polarization. In this way, we directly certify the relation between the light polarization and its momentum. Particularly analyzing *S*_3_(*θ*, *φ*), the link between the propagation direction of the light, and thus its momentum, to its spin (circular polarization) is determined. Here, the angle-resolved patterns of the Stokes parameter *S*_3_ reveal that the right- and left-handed circularly polarized lights are emitted in opposite directions. This, as we demonstrate later in the paper using both dark-field and cathodoluminescence polarimetry, is a direct consequence of the spin-momentum locking effect (QSHE of light). Our experiments provide new insights into the scattering behaviour of surface plasmon waves and their transverse spin angular momentum. Specifically, our results confirm that the scattering direction of the plasmonic waves is determined by the inherent transverse spin angular momentum carried by the waves, which is locked to the direction of surface plasmon propagation. These findings have significant implications for understanding the giant photonic spin hall effect associated with plasmonic materials, which has recently gained significant interest in the scientific community [29].

With a subwavelength shift of the electron impact position from a position in which the plasmonic crystal is viewed centrosymmetric to another point where the structure lacks inversion symmetry, a splitting is observed in the angle-resolved CL patterns. The site-selective emergence of the mode splitting provides a unique feature of the angle-resolved CL polarimetry that cannot be achieved via dark-field measurements. This is due to an efficient site-selective excitation of both modes of different parities via the localized electron beam excitations. The energy-momentum dispersion of the plasmonic crystal measured by cathodoluminescence spectroscopy and the numerically calculated band diagram both confirm the emergence of SOC-induced band splitting in our presented plasmonic crystal. The angular momentum vorticities calculated for each of the split modes show rotating behaviors in opposite directions due to the spin–orbit interactions in this structure.

Our research aims to investigate the potential of manipulating spin–orbit interactions by tailoring the building blocks of a plasmonic crystal. Plasmonic waves naturally possess spin-momentum interactions resulting from the spin component being orthogonal to their linear momentum, where the latter is aligned to their direction of propagation. By breaking the symmetry of the system or introducing an anisotropy, additional SOC effects like Rashba-type and Zeeman-type spin–orbit splitting can be induced. Our plasmonic crystal sustains a chiral configuration in each lattice element, which can effectively modify the angular momentum distribution in a three-dimensional basis, not only planar as investigated before via planar metamaterials leading to the optical Rashba effect [9, 10]. This aspect of three-dimensional chirality at the interface leads to a three-dimensional angular momentum profile for the plasmonic optical modes with components of both spin and angular momenta being parallel to each other, as will be later described here, leading to a bandgap in the energy-momentum dispersion diagram. Here, we seek to elucidate the underlying physics of these effects and assess their potential applications for the development of electronic and optoelectronic devices.

## 2 Results

**Figure 2:**
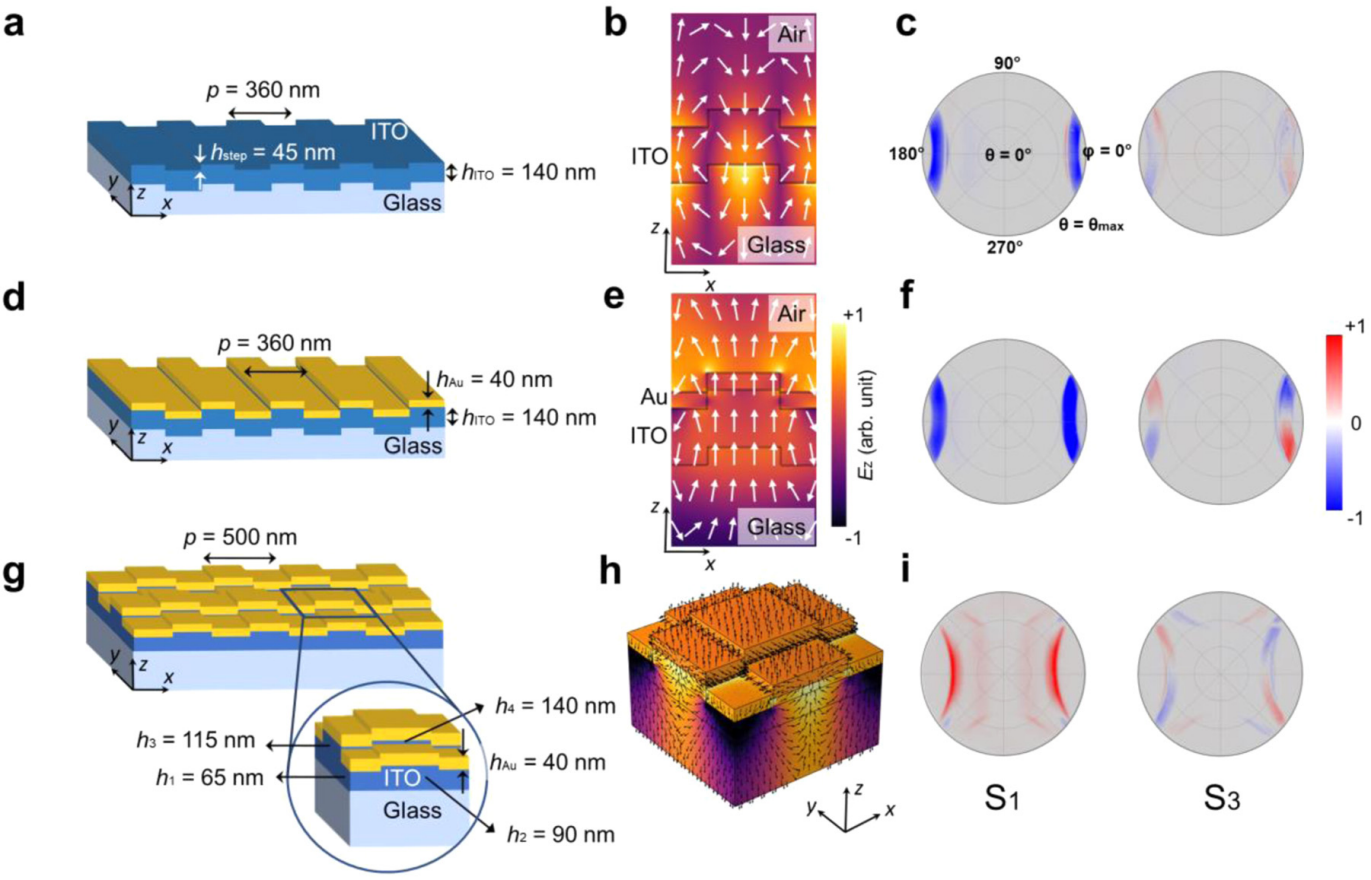
Spin-momentum locking of evanescent waves in photonic and plasmonic crystals. (a, d, g) Schematic representation of (a) 1D ITO grating patterned on a glass substrate, (d) Au-coated 1D ITO grating patterned on a glass substrate, and (g) 2D plasmonic crystal. The inset in (g) indicates the zoomed in view of a unit cell of the proposed 2D plasmonic crystal. (b, e, h) Spatial distribution of the *z*-component of the electric field (*E*_*z*_) within a single unit cell of the nanostructures shown in panels (a, d, g) calculated at *λ*_0_ = 550 nm (*E* ≈ 2.25 eV) in (b, e) and *λ*_0_ = 600 nm (*E* ≈ 2.07 eV) in (h). The arrows show the electric field polarization at each position. (c, f, i) Stokes parameters *S*_1_ and *S*_3_ corresponding to the structures shown in panels (a, d, g), measured at *λ*_0_ = 550 nm (*E* ≈ 2.25 eV) in (c, f) and *λ*_0_ = 600 nm (*E* ≈ 2.07 eV) in (i). *θ*_max_ is determined by the numerical aperture of the objective lens utilized in the dark-field measurements.

In order to investigate the behavior of the plasmonic crystal systematically, we provide a comparison between the following cases: (i) 1D ITO grating patterned on a glass substrate (Figure 2a), (ii) Au-coated 1D ITO grating patterned on a glass substrate (Figure 2d), and (iii) the proposed 2D plasmonic crystal (Figure 2g). In case (i), a 140-nm thick ITO layer is patterned to form a 1D grating with the periodicity of *p* = 360 nm and the step height of *h*_step_ = 45 nm. To create the structure (ii), a thin film of gold (*h*_Au_ = 40 nm) is deposited on the realized 1D ITO grating. The latter structure, i.e. the 2D plasmonic crystal, is created by two consecutive etching steps with different depths in perpendicular directions followed by the deposition of a 40-nm thick Au layer on the patterned ITO (see the Supplementary Information for the details of the fabrication method) [30]. The periodicity of the 2D plasmonic crystal is *p* = 500 nm. As illustrated in the inset of Figure 2g, each unit cell of the realized 2D plasmonic crystal consists of four sectors with different ITO heights (*h*_1_ = 65 nm, *h*_2_ = 90 nm, *h*_3_ = 115 nm, *h*_4_ = 140 nm) coated with gold. We numerically calculated the spatial distribution of the *z*-component of the electric field (*E*_*z*_) and measured the Stokes parameters with Dark-field polarimetry to analyze the polarization state of the light that is scattered from the aforementioned structures.

### 2.1 Electric field calculation

The *z*-component of the electric field (*E*_*z*_) within a single unit cell of the structures (i)–(iii) are shown in Figure 2b, e, and h, overlaid with arrows indicating the electric field polarization at each position. In samples (i) and (ii), since the structure is homogenous in *y*-direction, the electric field is calculated in *x*–*z* plane to reduce the computation time. The 1D grating couples the incident light to a partially evanescent and partially propagating wave, i.e. the waveguide mode of the ITO layer (in case (i)) and the surface plasmon mode at the interface of Au and ITO (in case (ii)), both propagating along the spatial modulation of the grating, i.e. ±*x*-direction, and with an evanescent tail along the *z*-direction. For a wave that propagates along +*x*-direction and decays exponentially along +*z*-direction, one can generally write the electric field as follows (see the Supplementary Information for the field calculation details) [23, 31]:

(6) $$E=\frac{A}{\sqrt{1+{\left|m\right|}^{2}}}\left(-im\frac{\kappa }{k_{x}}\bar{x}+\frac{k}{k_{x}}\bar{y}+\bar{z}\right)\exp\left(ik_{x}x-\kappa z\right)$$

Here $k=k_{x}\bar{x}+i\kappa \bar{z}$ is the complex wavevector, whereas *k*_*x*_ and $\kappa =\sqrt{k_{x}^{2}-k^{2}}$ are the longitudinal wave number along *x*-direction and the exponential decay rate along *z*-direction, respectively. $\bar{\alpha }$ with *α* ∈ (*x*, *y*, *z*) is the unit vector along the specified direction. The complex number *m*, as defined in the Supplementary Information, describes the polarization state of the wave. In contrast to propagating waves in free space with pure transverse polarizations, the wave described by Equation (6) possesses an imaginary longitudinal electric field component that induces rotation of the electric field in *x*–*z* plane resulting in an extraordinary transverse spin [31, 32] that can be written as [23]:

(7) $$S_{⊥}=\frac{Rek\times Imk}{{\left(Rek\right)}^{2}}$$

As opposed to the longitudinal SAM, the transverse SAM is orthogonal to the wavevector of the propagating wave and, interestingly, the direction of the transverse SAM is locked to the propagation direction of the wave [23, 33], [34], [35]. As can be observed in Figure 2b and e, the electric field rotates within the *x*–*z* plane, thereby generating a transverse SAM along the orthogonal *y*-direction. The spin-momentum locking phenomenon is more visible in the 3D field profile of the plasmonic crystal presented in Figure 2h, where oppositely propagating waves (+*x* and −*x*) carry transverse SAMs with opposite directions (+*y* and *−y*) and orthogonally propagating waves (+*x* and +*y*) possess SAMs in orthogonal directions (+*y* and *−x*). This property of the propagating waves with evanescent tails will become more obvious by measuring the polarization states of the scattered light from the structures, as it is described below.

### 2.2 Dark-field polarimetry

When an evanescent wave propagates in a certain direction (e.g. +*x*), it is scattered to the free space upon interaction with the periodic structure. Owing to the non-zero transverse SAM of the evanescent wave, the scattered light is decomposed into opposite circular polarizations (RCP and LCP) emitting in opposite directions (*−y* and +*y*). An evanescent wave propagating in the *−x* direction, on the other hand, carries a transverse SAM in the opposite direction. Thus, directional splitting of the RCP and LCP components of the scattered light can be observed in the opposite direction (+*y* and *−y*). To gain a better understanding of the spin-momentum locking phenomena, we analyzed the polarization state of the light scattered from each of the previously mentioned structures via dark-field polarimetry. For this purpose, we placed a quarter wave plate (QWP) followed by a linear polarizer at the detection path of a dark-field microscopy setup and measured the Stokes parameters for each sample (see Methods for more details about dark-field polarimetry).

The angular patterns of *S*_1_ and *S*_3_ Stokes parameters corresponding to the different samples are presented in Figure 2c, f, and i. The Stokes parameters obtained from the 1D gratings (Figure 2c and f) demonstrate that light is coupled to the guided transverse electric (TE), i.e. polarized along the *y*-axis or *φ* = 90°, modes propagating along ±*x*-direction with clear splitting of the RCP and LCP components (indicated by red and blue colors in *S*_3_, respectively). Comparison of the Stokes parameters obtained from the 1D ITO grating without/with Au (Figure 2c and f) reveals that guided modes in both samples exhibit a similar spin-dependent direction splitting behavior. Nevertheless, excitation of surface plasmon waves in the latter case (Figure 2f) enhances the intensity of the scattered light and emphasizes the splitting.

In the 2D plasmonic crystal, light is mostly coupled to transverse magnetic (TM), i.e. polarized along the *x*-axis or *φ* = 0°, surface plasmon waves that propagate along orthogonal in-plane directions, i.e. ±*x* and ±*y*. However, an almost directional propagation of the surface plasmon waves is observed in the corresponding angle-resolved Stokes parameters (Figure 2i), where the modes propagating along *x*-direction are more visible than those propagating in *y*-direction. To better understand the origin of this nearly directional propagation, one has to notice the fabrication process of the sample (explained in the Supplementary Information), wherein an ITO layer was first etched to form a 1D grating with spatial modulation along *x*-direction and a groove height of 25 nm. Then, a second round of etching was carried out along *y*-direction with a different etching depth of 50 nm. As a result, the groove height of the realized 2D ITO grating along *y*-direction is not of the same value as that along *x*-direction ( $h_{g}^{x}$ = 25 nm, $h_{g}^{y}$ = 50 nm). Deposition of a 40-nm thick Au film on the resulting 2D ITO grating produces a 2D plasmonic structure in which the metallic layer is continuous along *x*-direction, whereas formation of discontinuities along *y*-direction hinders the propagation of surface plasmon waves in the latter direction. The angle-resolved pattern of the *S*_3_ Stokes parameter (Figure 2i) shows spin-dependent direction splitting of the modes propagating either along *x* or *y* direction. One may notice that the periodicities of the 1D and 2D lattices are slightly different. This causes a slight shift of the diffraction rings in the angle-resolved patterns and does not affect the physical properties we investigate, and particularly the spin–orbit interactions in our structures. The measurements here were based on the RGB camera installed in our setup for Fourier imaging, thus accessing to the behavior of the structures in the longer wavelength ranges were not possible. Hence, we use CL spectroscopy to gain more insight into the broadband and site-specific behaviors of the 2D plasmonic crystal.

### 2.3 Cathodoluminescence spectroscopy and polarimetry

In cathodoluminescence spectroscopy, the sample is excited by a high-energy electron beam produced in a scanning electron microscope (SEM) chamber and the light emitted from the sample is then collected and analyzed [36, 37]. Owing to localized excitation of optical modes by an electron beam, CL spectroscopy offers a wider spectral range and a superior spatial resolution in comparison to dark-field spectroscopy and other optical methods. While the maximum spatial resolution that can be achieved by standard optical methods is limited by the diffraction of light, electron-based techniques can reach nanometer resolutions. This extraordinarily high spatial resolution enables us to selectively excite the localized modes in samples with spatially varying properties.

In cathodoluminescence, the electron beam is classically described as a current density of $j\left(\rho ,t\right)=-e\delta \left(\rho -\rho_{0}\right)\delta \left(z-v_{e}t\right)$ , where *e* and *v*_*e*_ are the charge and velocity of electron and **ρ** gives the impact position of the electron beam on the sample [38]. When an electron impinges on a metal, it induces an electric dipole oriented normal to the surface (*E*_z_) that produces surface plasmon waves propagating in all azimuthal angles. Upon interacting with the periodic lattice, the plasmonic waves are then coupled to out-of-plane radiating modes that can be collected in the far-field [39]. The spatial and spectral properties of the plasmonic modes resolved by CL measurements are related to the radiative local density of states (LDOS) of the sample. More precisely, it has been demonstrated that the CL emission is related to the radiative LDOS projected along the electron-beam trajectory [40, 41].

**Figure 3:**
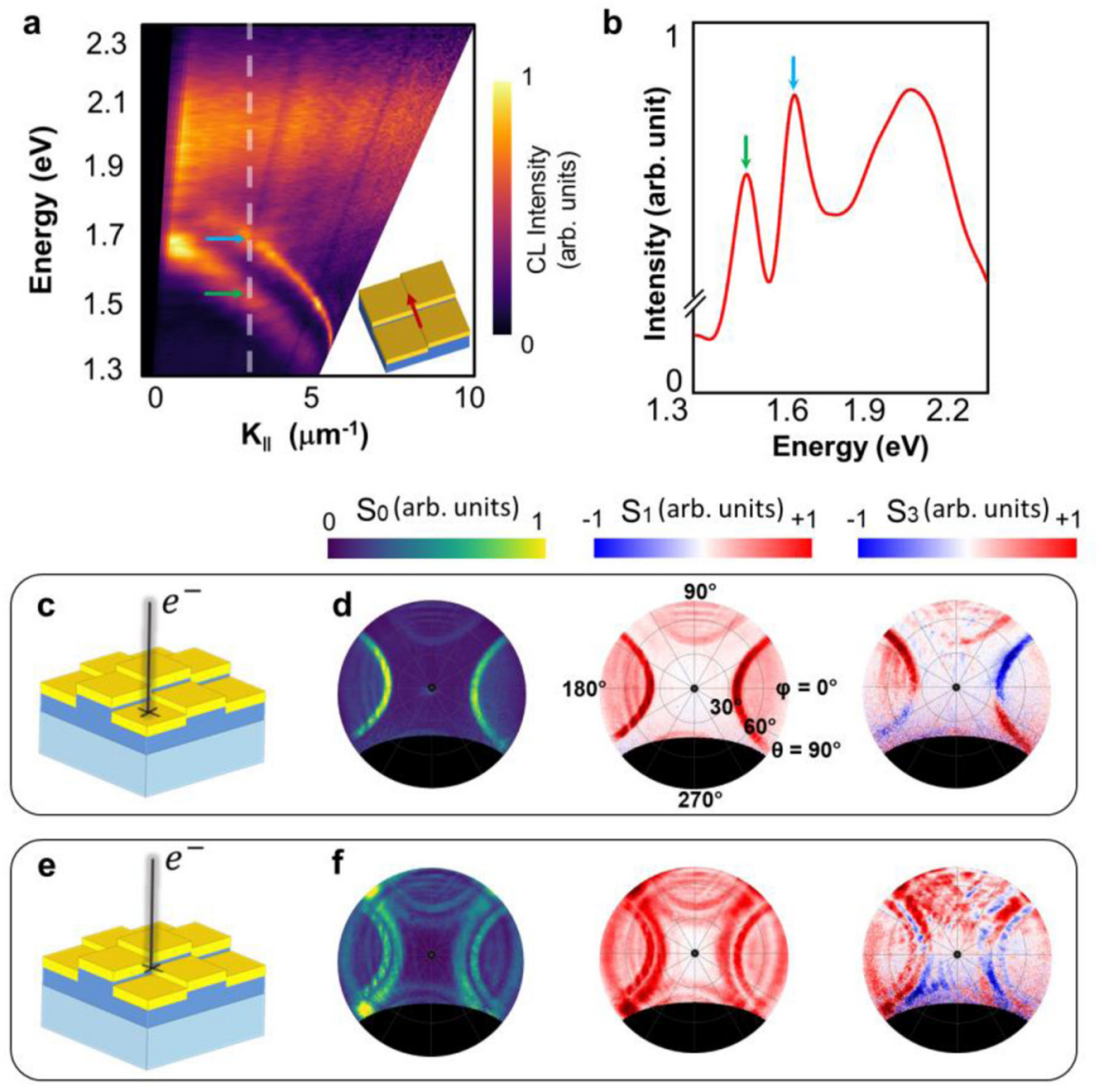
Measured cathodoluminescence energy-momentum map, demonstrating the energy splitting of the plasmonic dispersion line. (a) Nondegenerate energy-momentum CL map measured along the direction indicated by a red arrow in the inset. (b) CL spectrum in a constant wavevector indicated by dashed lines in panel (a). The spectral positions of the peaks are indicated by colored arrows. (c, e) Schematic illustration of the sample excited by an electron beam at different positions: (c) at sector 1 of a unit cell, where the ITO thickness is *h*_1_ = 65 nm and (e) at the crossing point of the four sectors of a unit cell. (d, f) The Stokes parameters *S*_0_, *S*_1_ and *S*_3_ measured at *E* = 1.55 eV (*λ* ≈ 800 nm) for the electron impact positions indicated in panels (c) and (e), respectively.

To gain better insight into the optical behavior of the proposed plasmonic crystal, we have measured the energy-momentum dispersion of the CL emission from the sample along the direction indicated by a red arrow in the inset of Figure 3a. The spot size of the electron beam is fixed to cover the whole unit cell of the structure. The dispersion diagram is acquired by placing a 1D slit in the optical path of the CL emission to select a specific azimuthal angle and a range of polar angles from 0 to 90°. The filtered CL light is dispersed by means of a grating onto the screen of a CCD camera [42]. The energy-momentum dispersion map shows a clear energy band splitting within a broad range of momentum and energy values (*E* < 1.8 eV, *λ* > 689 nm). Note that the dark zone in the low-*k* region of the energy-momentum CL map in Figure 3a is due to the hole inserted in the hyperbolic mirror, to allow the electron beam to pass through the mirror. We also plotted the CL spectrum at a fixed in-plane wavevector that is indicated by a vertical dashed line on the energy-momentum map and marked the spectral positions of the two peaks by colored arrows (Figure 3b). We attribute this band splitting phenomenon, wherein two parallel bands are observed over a wide range of energy and momentum, to the SOC-induced angular momentum redistribution of the light emitted from the structure. In plasmonic systems, splitting of the modes may occur as a result of plasmon hybridization [43]. In our proposed structure, we observe no hybridization of photonic and/or plasmonic modes in our energy range of interest wherein the band splitting appears (*E* < 2 eV). To demonstrate this, we analytically calculated the dispersion of the photonic and plasmonic modes in three different structures: (1) an ITO layer with a fixed thickness, (2) a 40-nm-thick Au layer, and (3) an Au-coated ITO layer. To assess the influence of the ITO layer thickness on photonic and plasmonic modes dispersion, we carried out the calculations for different thicknesses of the ITO layer (*h*_1_ = 65 nm, *h*_2_ = 90 nm, *h*_3_ = 115 nm, *h*_4_ = 140 nm). The results show no evidence of photonic and/or plasmonic modes hybridization in the energy range of *E* < 2 eV. The dispersion diagrams acquired for the aforementioned cases with different thicknesses of the ITO layer are presented in Figure S7. In the followings, we will show that the differences in these two optical modes are due to the differences in the angular momenta of light.

To investigate the polarization state of the CL emission from the sample, we determine the Stokes parameters *S*_0_, *S*_1_ and *S*_3_ by using the angle-resolved CL polarimetry setup demonstrated in Figure 1b. The Stokes parameters were acquired at two different electron impact positions indicated schematically in Figure 3c and e: (1) where the ITO thickness is *h*_1_ = 65 nm (Figure 3c) and (2) at the crossing point of the four sectors with different ITO heights (Figure 3e). The resulting CL emission is filtered at a certain energy *E* = 1.55 eV (*λ* ≈ 800 nm). When the electron beam traverses the sample at position 1 (Figure 3c), the measured Stokes parameters exhibit spin-dependent direction splitting of the surface plasmon modes propagating along *x* or *y* direction (Figure 3d), which is in total agreement with the results achieved by the dark-field polarimetry method. Considering the mode propagating in the +*x*-direction, demonstrated by the semicircle at the right side of the angular *S*_3_ pattern, the state of circular polarization (spin) flips sign by altering the azimuthal angle *φ* at a certain polar angle, e.g. *θ* = 60°. We ascribe this azimuthal spin-splitting behavior to the spin-momentum locking phenomenon in plasmonic waves. As mentioned before, this phenomenon is a universal property of the plasmonic waves and appears regardless of the symmetry condition of the system. Excitation of the sample at the crossing point of the four sectors with different ITO heights (Figure 3e), on the other hand, leads to the emergence of two non-degenerate modes as previously demonstrated in Figure 1a. It can be observed clearly in the Stokes parameters *S*_0_, *S*_1_ and *S*_3_ angle-resolved patterns (Figure 3f) that an additional splitting of the modes propagating in the *x* and *y* directions appears. A closer look at the *S*_3_ parameter provides further information about the state of circular polarization (spin) of the split modes. By increasing the polar angle *θ* at a fixed azimuthal angle, e.g. *φ* = 30°, the state of circular polarization (spin) of the emitted light changes from right-handed to left-handed, shown by red and blue colors respectively. This polar splitting of the modes only occurs when the electron beam traverses the sample at the crossing point of the four sectors (Figure 3e). In this peculiar point, the electron beam couples to two different modes of the structure that as will be discussed later, have different parities and undergo an intriguing spin–orbit interaction, demonstrated by their different vorticities as well.

Indeed, the impact position of the electron on the sample significantly affects the angular momentum distribution of the emitted light and the resulting spin–orbit interactions. Thus, positioning the electron beam on sites 1 and 2, the angle-resolved CL maps filtered at the energy of *E* = 1.55 eV (*λ* ≈ 800 nm) show quite different behaviors. As it will be shown in the following section, the spin–orbit interactions break the degeneracy of the plasmonic Bloch modes producing two symmetric and antisymmetric field distributions with different spin vorticities. Thus, site-selective excitations of the sample with the electron beam allow us to decompose the modes. When the electron traverses the sample at the impact position shown in Figure 3c, it excites only the symmetric configuration, while the excitation at the position shown in Figure 3e, could resolve both modes.

### 2.4 Numerical simulations

**Figure 4:**
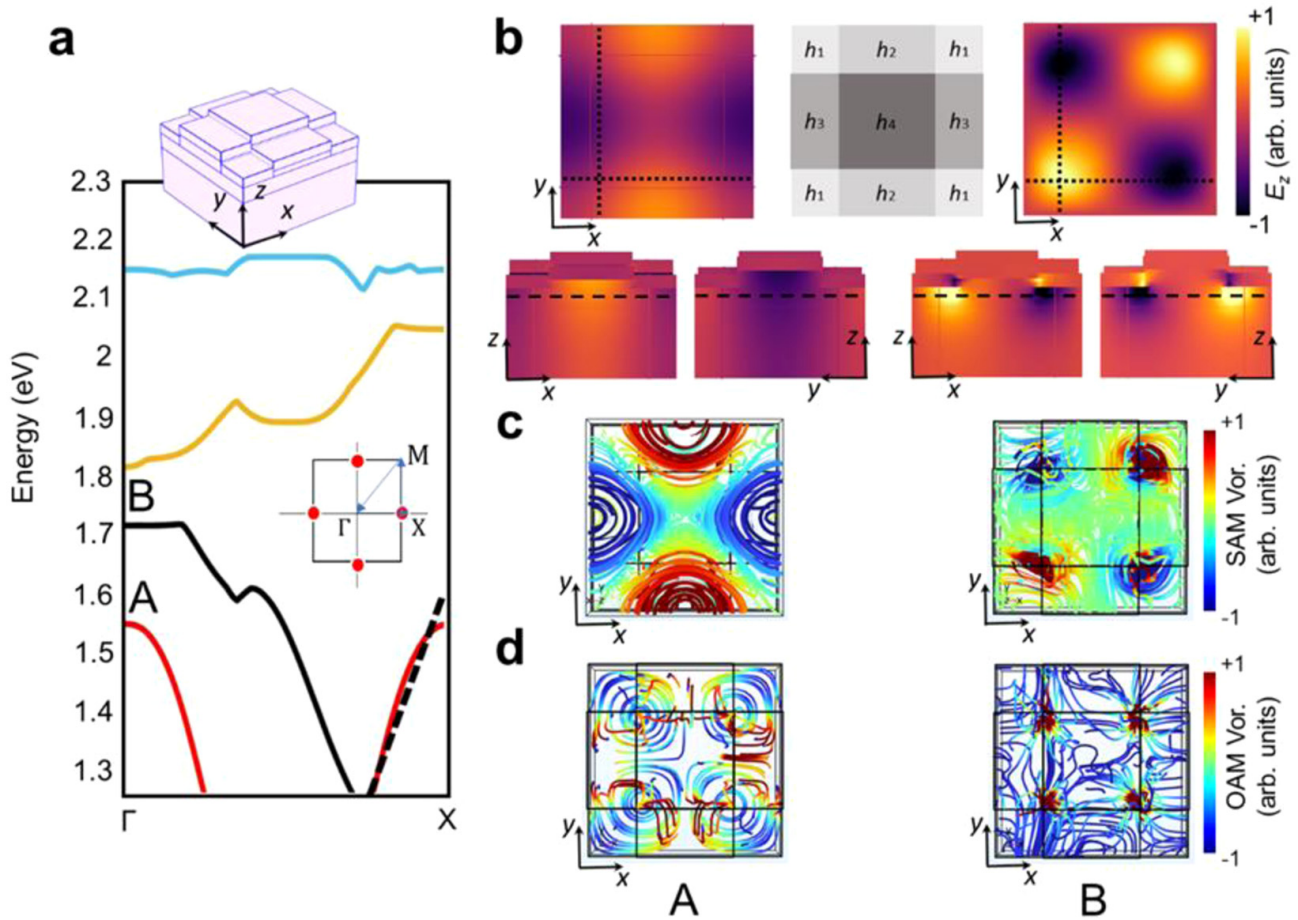
Simulated band diagram. (a) The band structures of the plasmonic crystal calculated along Γ–*X* direction in the reciprocal space. Black dashed line indicates the light line. The insets show unit cells of the corresponding simulated crystals. (b) Top view and cross-sections of the electric field (*E*_z_) calculated at points A (left column) and B (right column) shown in the band structure. The schematic diagram indicates the thicknesses of ITO layers at each region of the unit cell. The dotted and dashed lines indicate the planes in which the cross-sections and top profile are acquired. (c) Spin and (d) orbital angular momentum vorticities calculated at points A (left column) and B (right column) shown in the band structure.

To better understand the origin of this broken degeneracy, the photonic band diagram of the proposed plasmonic crystal is numerically calculated using COMSOL Multiphysics software and compared with the experimentally measured energy-momentum dispersion map. The band diagram for Γ–*X* path of the first Brillouin zone is depicted in Figure 4a (see Methods for the details of the numerical simulation), which is in good agreement with the results obtained from the CL measurements (Figure 3a). In the measured energy-momentum map, higher energy modes are manifested as a continuum of optical modes covering a broad range of energies. This is due to the large decay rate of these optical modes associated with the onset of the d-Band transitions in gold. Since the decay rates of the optical modes are not considered in our simulations, the higher energy modes appear as discrete bands in Figure 4a. SOC effects appear in the form of splitting of the energy bands that is clearly observed for the two low-energy modes at Γ point.

To compare the optical response of the system at the two different modes, we calculated the *z*-component of the electric field at points A and B indicated in the bandstructure. Figure 4b exhibits the resulting electric field profiles for A (left panel) and B (right panel) displayed in three different views: (i) *z*-normal plane located below the ITO/Au interface indicated by the dashed lines, (ii) *x*-normal and (iii) *y*-normal planes indicated by the dotted lines in Figure 4b. While the electric field of the first mode penetrates into the substrate, the second mode is highly confined to the ITO/Au interface. Furthermore, the field distribution in the latter case is antisymmetric showing opposite polarities at the two sides of the ITO/Au interface.

The value of band splitting Δ*E* is related to the scalar product of the orbital and spin parts of the total angular momentum:

(8) $$\Delta E\left(k\right)=A_{SO}\left(k\right)L\cdot S$$

Here, the values of band splitting Δ*E* and spin–orbit parameter *A*_SO_ are functions of the wavevector *k*. There are different approaches to define and connect the spin and orbital angular momentum of light. For example, Vuong et al. proposed analytical relations to study of the spin–orbit interactions in 2D structures [44]. Here, we use the principles and concepts introduced by M.V. Berry [45]. One can write the total angular momentum as the sum of spin and orbital components:

(9) $$J=S+L=\int R\times \left(p_{S}+p_{O}\right)dR$$

where **p**_**S**_ and **p**_**O**_ are the spin and orbital flow densities given by:

(10) $$p_{S}=-\frac{c\varepsilon_{0}}{8\omega }Im\left[\nabla \times \left(E^{*}\times E+H^{*}\times H\right)\right]$$

(11) $$p_{O}=\frac{c\varepsilon_{0}}{4\omega }Im\left[\left(E^{*}\cdot \nabla \right)E+\left(H^{*}\cdot \nabla \right)H\right]$$

For each wavevector *k* along the Γ–*X* direction, we compute the value of **L** ⋅ **S** numerically, attain the value of Δ*E* from the band diagram of Figure 4a and, thereby, estimate the spin–orbit parameter *A*_SO_(*k*) using Equation (8). The variation of the resulting spin–orbit parameter *A*_SO_ as a function of the wavevector *k* is demonstrated in Figure S3. One can expand the scalar product of **L** ⋅ **S** using basic vector algebra and demonstrate extraordinary terms appearing in the form of **E** ⋅ H* in addition to the ordinary quadratic energy terms in the forms of **E** ⋅ E* and **H** ⋅ H* (see Supplementary Information for the details). This behavior can be described by the Axion electrodynamics equations, wherein emergence of extra energy terms in the Hamiltonian gives rise to a peculiar angular momentum distribution and therefore degeneracy breaking of optical modes.

To visualize the rotational flow of angular momentum in our proposed structure, we computed the total angular momentum vorticity of light that is the sum of the spin angular momentum (SAM) and the orbital angular momentum (OAM) vorticities [45, 46]:

(12) $$\Omega =\Omega_{S}+\Omega_{O}$$

The SAM and OAM vorticities are:

(13) $$\Omega_{S}=\nabla \times p_{S}=-\frac{c\varepsilon_{0}}{8\omega }Im\left[\nabla^{2}\left(E^{*}\times E+H^{*}\times H\right)\right]$$

(14) $$\Omega_{O}=\nabla \times p_{O}=\frac{c\varepsilon_{0}}{4\omega }Im\left[\nabla E^{*}\cdot \left(\times \nabla \right)E+\nabla H^{*}\cdot \left(\times \nabla \right)H\right]$$

In Equation (14), the scalar product links the field vectors, whereas the vector product relates the gradient operators ∇. Therefore, using the convention specified in Ref. [40], we can write:

(15) $$\nabla E^{*}\cdot \left(\times \nabla \right)E=\nabla E_{x}^{*}\times \nabla E_{x}+\nabla E_{y}^{*}\times \nabla E_{y}+\nabla E_{z}^{*}\times \nabla E_{z}$$

The spin and orbital angular momentum vorticities are calculated at the points A and B indicated in Figure 4a. The top views of the SAM vorticities calculated for the first and second modes (Figure 4c) conform well to the field distributions of the corresponding modes, exhibiting rotational flow around the electric field extrema. The spin rotational flow is shown to be right (left)-handed around the maxima (minima) of the electric field. The color map in Figure 4c depicts the size and the direction of the SAM flow, with red and blue denoting right- and left-handed rotations, respectively. The cross-sectional view of the spin and orbital angular momentum vorticities calculated at the points A and B are shown in Figure S4 to provide a better understanding of the SAM and OAM orientations. The total angular momentum vorticity at point A (Figure S5, top panel) reveals apparent rotation that is nearly in opposite direction comparing to that calculated at point B (Figure S5, bottom panel). The opposite flow of the total angular momentum vorticities obtained for the two split modes conveys the idea that the SOC effects play a significant role in the emergence of the band splitting observed in the proposed plasmonic crystal.

Finally, to provide a better understanding of the influence of the electron impact position on angular momentum of the emitted light, we calculated the spin, orbital and total angular momentum spatial distributions when the sample was excited by an electron beam at two different sites shown in Figure 3c and e: (1) where the ITO thickness is *h*_1_ = 65 nm and (2) at the crossing point of the four sectors with different ITO heights. Our simulation results, shown in Figure S6, confirm our claim that the excitation position has a significant impact on the distribution of the SAM and OAM in the emitted light and the resulting spin–orbit interactions. While the SAM and OAM induced in the former case are nearly orthogonal leading to a minimum value of **L** ⋅ **S**, the projection of spin on orbital angular momentum in the latter case becomes maximum giving rise to an enhanced spin–orbit interaction.

## 3 Conclusions

Spin–orbit interactions, i.e. the coupling of spin and orbital angular momenta of light, crucially affects the behavior of light at the subwavelength scales. One can manipulate the spin–orbit coupling effects by deliberate arrangement of nanostructures in a photonic or plasmonic crystal in order to bring forth novel functionalities and applications in nanooptics. Herein, by combining the experimental results obtained from the cathodoluminescence spectroscopy with those of numerical simulations, we have demonstrated that the spin–orbit coupling effects in a plasmonic crystal can induce splitting of the energy bands giving rise to non-degenerate plasmonic modes with opposite angular momentum distributions. The SOC effects was shown to be strongly site-selective, occurring only when the electron beam excites the surface modes at the crossing point of the sectors with different ITO heights, wherein optical modes of various symmetries can be excited.

Moreover, we determined the polarization state of the light emitted from the sample using angle-resolved CL and dark-field polarimetry. The angular patterns of the Stokes parameters acquired from both methods indicated a polarization-to-direction−coupled emission from the plasmonic crystal. This is attributed to QSHE of light through which the surface plasmon waves propagating in opposite directions carry opposite transverse SAM. Interacting with the periodic crystal, these propagating surface modes scatter to the far-field in different directions based on their polarization (spin) state. This is evident from the angular patterns of the *S*_3_ Stokes parameter with a clear spin-dependent direction splitting of the modes. To provide a more precise analysis of the phenomena, we also measured the Stokes parameters for 1D ITO gratings without/with Au layer and compared the results with those obtained from the 2D plasmonic crystal. The fact that a similar spin-dependent direction splitting appears in all samples manifest that the QSHE is not dependent on the symmetry condition of the structure and is intrinsic to all modes with evanescent tails, including waveguiding and surface modes.

Our numerical simulations revealed a band splitting in the *E*–*k* diagram of the plasmonic crystal. We argued that the band splitting value (Δ*E*) is linked to the value of **L** ⋅ **S** via the wavevector-dependent spin–orbit parameter *A*_SO_. To shed light on the connection between the angular momentum components and the energy splitting, we analytically derived the scalar product of the spin and orbital components of the angular momentum to show extraordinary **E** ⋅ H* terms appearing in the Hamiltonian of system in addition to the conventional quadratic energy terms. We attribute the Zeeman-type band splitting occurring in our plasmonic structure to these extra terms.

By calculating the electric field profiles related to the split modes at their Γ points, we demonstrated the formation of symmetric and antisymmetric plasmonic modes with different field distributions. These symmetric and antisymmetric modes could be excited selectively by site-selective excitation of the sample with the electron beam. Besides, we conducted numerical simulations to acquire the vorticities of the spin, orbital and total angular momentum in the proposed plasmonic crystal. The total angular momentum vorticities acquired for the two split modes showed rotational behavior with opposite directions. Both SAM and OAM contribute to the total angular momentum distribution leading to the formation of the SOC-induced band splitting, as revealed in the energy-momentum dispersion diagrams obtained from CL measurements and numerical simulations. In contrast to the QSHE of light that is an inherent property of any waveguiding or surface wave, occurrence of band splitting depends on the symmetry status of the studied sample at the excitation position. Our work may be the first to experimentally demonstrate both effects in plasmonic crystals and can open new pathways for the design of novel spin–orbit plasmonic devices.

## 4 Methods

### 4.1 Angle-resolved dark-field polarimetry

The dark-field polarimetry was carried out using an inverted microscope (Nikon Eclipse Ti2-A) integrated in a NanoMicroSpec-Transmission New Technologies and Consulting Setup. A specialized dark-field condenser enabled diascopic dark-field illumination, i.e. light was only focused onto the sample in an annular ring of large incident angles while the objective was located on the opposite side of the sample. The applied objective was a Nikon S Plan Flour ELWD Series objective with a 60× magnification and a numerical aperture of 0.7, which was smaller than the numerical aperture range of the condenser lens (0.8–0.95). Consequently, only light redirected or emitted by the sample was collected by the condenser. To gain the Stokes parameters, a quarter-wave plate was placed behind the objective followed by a linear polarizer which filtered the collected light by circular or linear polarization, depending on the orientations of their optical axes. Angle-resolved images were obtained by inserting an extra lens, commonly called “Bertrand lens”, into the optical path behind the image plane. It focused light rays with the same incident angle in the image plane into one spot of a plane and therefore created a new Fourier image. Optical band pass filters were used to filter the angle-resolved images captured by an Allied Vison Prosilica GC 2450C camera which is an RGB camera optimized for the visible range of light.

### 4.2 CL spectroscopy and polarimetry

To perform CL spectroscopy, we used an optical field emission microscope (Zeiss SIGMA) that was equipped with CL compartment made by Delmic B.V. A focused electron beam with an acceleration voltage of 30 keV and a beam current of 11 nA was utilized to excite the surface of the sample. Emitted light was then collected by an off-axis Aluminum parabolic mirror positioned above the sample and was directed to a CCD camera to produce angle-resolved maps (as shown in Figure 1b). The mirror had a focal distance of 0.5 mm, an acceptance angle of 1.46*π* sr, and dwell time of 35 ms. Angle-resolved patterns formed after the optical beam passed through a color filter to select a specific wavelength (bandwidth of 50 nm). To study the polarization state of the CL emission, we exploited an additional polarizer module including a quarter wave plate (QWP) and a linear polarizer with transmission axes adjusted at angles *α* and *β*, respectively. The angle-resolved CL intensity patterns *I*_*j*_ are measured for six different combinations of (*α*, *β*) and the Stokes parameters were calculated by [47]:

$$S_{0}=I\left(0{}^{\circ},0{}^{\circ}\right)+I\left(0{}^{\circ},90{}^{\circ}\right)$$

(16) $$S_{1}=I\left(0{}^{\circ},0{}^{\circ}\right)-I\left(0{}^{\circ},90{}^{\circ}\right)$$

$$S_{2}=I\left(0{}^{\circ},45{}^{\circ}\right)-I\left(0{}^{\circ},-45{}^{\circ}\right)$$

$$S_{3}=I\left(45{}^{\circ},0{}^{\circ}\right)-I\left(-45{}^{\circ},0{}^{\circ}\right)$$

where *S*_0_ describes the total intensity of the CL emission, *S*_1_, *S*_2_ and *S*_3_ correspond to the linear, diagonal and circular polarization of the light, respectively.

To acquire high quality energy-momentum maps, we used a 220 µm wide 1D slit in the optical path of the CL emission to select a specific momentum component of the emitted light. Each energy-momentum CL measurement was carried out over a dwell time of 180 s and a diffraction grating was used to disperse the light on the camera [42].

### 4.3 Numerical simulations

We conducted numerical simulations using COMSOL Multiphysics software in order to shed light on the optical modes of the plasmonic lattice. The radiofrequency (RF) toolbox of COMSOL was used in a 3D simulation domain, which is based on solving Maxwell’s equations in real space and in the frequency-domain using the finite-element method. In order to calculate the band diagrams, a stationary solver was employed to solve a nonlinear eigenvalue problem with the normalized electric field as the eigenvalue. What one obtains with the nonlinear formulation is that the mode normalization performed by the global equation involves setting the domain integral of $E_{z}\cdot {E_{z}}^{*}$ to unity. The simulation domain was constrained into a single unit cell by the Floquet periodic boundary conditions, and the parametric solver swept the wavevector **k**. The reciprocal lattice vectors of the plasmonic crystal dictate the range of **k**.

## Supplementary Material

Supplementary Material Details
