# Supplementary material for: Spin–orbit interactions in plasmonic crystals probed by site-selective cathodoluminescence spectroscopy
Source: Nanophotonics. 2023 Apr 5;12(10):1877–89. doi: 10.1515/nanoph-2023-0065 (PMC10161781; doi:10.1515/nanoph-2023-0065)
Supplement: Supplementary file 1 — Supplementary Material Details [file j_nanoph-2023-0065_suppl_001.pdf]

Supplementary Information

Masoud Taleb, Mohsen Samadi*, Fatemeh Davoodi, Maximilian Black, Janek Buhl, Hannes Lüder, Martina Gerken, and Nahid Talebi*

Spin-orbit interactions in plasmonic crystals probed by site-selective cathodoluminescence spectroscopy

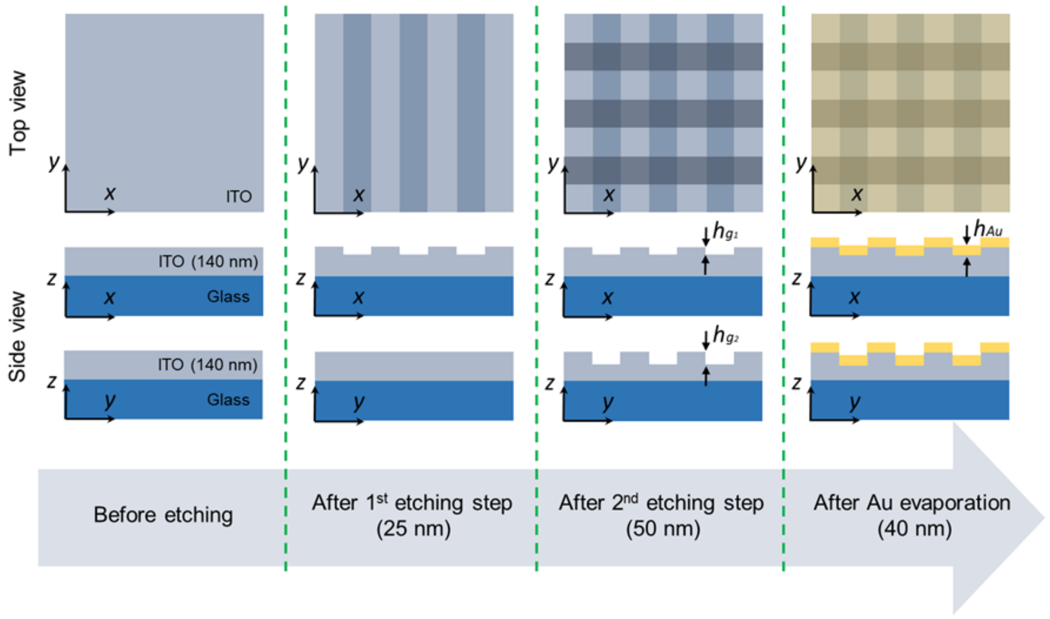

Figure S1: Schematic illustration of the fabrication method. A 140 nm thick ITO layer is deposited on a glass substrate. The ITO layer is etched using UV nanoimprint lithography to form a 1D grating along x-direction with a groove height of h_{g_1} = 25 nm. The sample is then etched along y-direction with an etching depth of h_{g_2} = 50 nm to form a 2D grating. Finally, the plasmonic crystal is built by evaporating a 40 nm thick layer of Au on the patterned ITO layer [1].

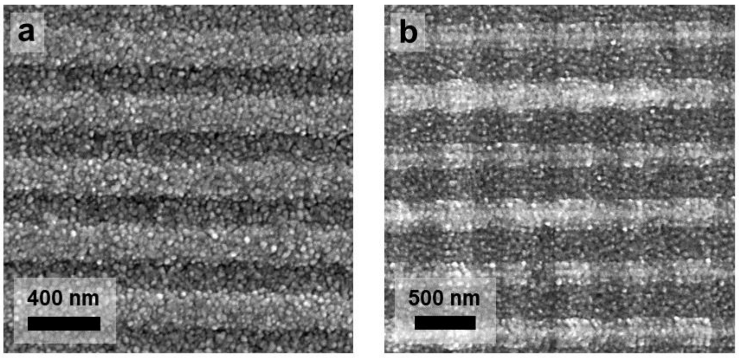

Figure S2: SEM images of the (a) Au-coated 1D ITO grating patterned on a glass substrate, and (b) 2D plasmonic crystal.

*Corresponding authors: Nahid Talebi,** Institute of Experimental and Applied Physics, Kiel University, 24098 Kiel, Germany; and Kiel, Nano, Surface, and Interface Science, Kiel University, 24098 Kiel, Germany, E-mail: talebi@physik.uni-kiel.de. https://orcid.org/0000-0002-3861-1005. **Mohsen Samadi,** Institute of Experimental and Applied Physics, Kiel University, 24098 Kiel, Germany, E-mail: samadi@physik.uni-kiel.de. https://orcid.org/0000-0001-6302-7612.

Masoud Taleb, Mohsen Samadi, Fatemeh Davoodi and Maximilian Black, Institute of Experimental and Applied Physics, Kiel University, 24098 Kiel, Germany

Janek Buhl, Hannes Lüder, Martina Gerken, Integrated Systems and Photonics, Faculty of Engineering, Kiel University, 24143 Kiel, Germany

Field calculation for the evanescent wave

First, we consider an electromagnetic wave propagating along *x*-direction in a medium with permittivity ε and permeability μ whose complex electric field can be written as [2,3]:

$$\mathbf{E} = \frac{E_0\sqrt{\mu}}{\sqrt{1+|m|^2}}\begin{pmatrix}0\\1\\m\end{pmatrix}e^{ikx} \tag{S1}$$

where E_0 and $k = n\omega / c$ denote the amplitude and the wavenumber of the electromagnetic wave and *n* is the refractive index of the medium. The polarization state of the wave can be expressed as functions of the complex number *m*:

$$\tau = \frac{1-|m|^2}{1+|m|^2}, \quad \chi = \frac{2Re(m)}{1+|m|^2}, \quad \sigma = \frac{2Im(m)}{1+|m|^2} \tag{S2}$$

Here, τ, χ, and σ are the normalized Stokes parameters exhibiting the degrees of linear, diagonal and circular polarizations respectively. Therefore, *m* = 0 and *m* = ∞ indicate the horizontal and vertical linear polarizations, *m* = $\pm$1 denote the diagonal and anti-diagonal polarizations and *m* = $\pm i$ correspond to the right-handed and left-handed circular polarizations.

One can obtain the electric field of an evanescent wave propagating along *x*-direction and decaying along *z*-direction by rotating the equation (S1) by an imaginary angle $i\varphi$ about *y*-axis. This transformation can be described by the matrix:

$$\mathbf{R}(i\varphi) = \begin{pmatrix}\cosh\varphi & 0 & -i\sinh\varphi\\ 0 & 1 & 0\\ i\sinh\varphi & 0 & \cosh\varphi\end{pmatrix} \tag{S3}$$

By applying the rotational transformation to the electric field described by the equation (S1), we have:

$$\mathbf{E} = \frac{E_0\sqrt{\mu}}{\sqrt{1+|m|^2}}\left(-im\frac{\kappa}{k_x}\bar{\mathbf{x}} + \frac{k}{k_x}\bar{\mathbf{y}} + \bar{\mathbf{z}}\right)e^{ik_x x-\kappa z} \tag{S4}$$

where $k_x = k\cosh\varphi$ and $\kappa = k\sinh\varphi$ are the propagation and decay constants respectively. The magnetic field of the evanescent wave can also be calculated using the same method.

Calculation of $\mathbf{p}_\mathrm{S}\cdot\mathbf{p}_\mathrm{O}$

As shown in equation (9), the total angular momentum of light is defined as the integration of (spin and orbital) angular momentum densities that are directly acquired from the (spin and orbital) flow densities $\mathbf{p}_\mathrm{S}$ and $\mathbf{p}_\mathrm{O}$. Here, we exploit equations (10) and (11) to derive the scalar product of the orbital and spin flow densities $\mathbf{p}_\mathrm{S}\cdot\mathbf{p}_\mathrm{O}$:

$$\mathbf{p}_\mathrm{S}\cdot\mathbf{p}_\mathrm{O} = -\frac{c^2\varepsilon_0^2}{32\omega^2}\mathrm{Im}\left\{\left[\nabla\times\left(\mathbf{E}^*\times\mathbf{E}+\mathbf{H}^*\times\mathbf{H}\right)\right]\cdot\left[\left(\mathbf{E}^*\cdot\nabla\right)\mathbf{E}+\left(\mathbf{H}^*\cdot\nabla\right)\mathbf{H}\right]\right\} \tag{S5}$$

The scalar product inside the curly brackets can be expanded as follows:

$$\begin{aligned}&\left[\nabla\times\left(\mathbf{E}^*\times\mathbf{E}+\mathbf{H}^*\times\mathbf{H}\right)\right]\cdot\left[\left(\mathbf{E}^*\cdot\nabla\right)\mathbf{E}+\left(\mathbf{H}^*\cdot\nabla\right)\mathbf{H}\right]=\\&\left[\nabla\times\left(\mathbf{E}^*\times\mathbf{E}\right)\right]\cdot\left[\left(\mathbf{E}^*\cdot\nabla\right)\mathbf{E}\right]+\left[\nabla\times\left(\mathbf{E}^*\times\mathbf{E}\right)\right]\cdot\left[\left(\mathbf{H}^*\cdot\nabla\right)\mathbf{H}\right]\\&+\left[\nabla\times\left(\mathbf{H}^*\times\mathbf{H}\right)\right]\cdot\left[\left(\mathbf{E}^*\cdot\nabla\right)\mathbf{E}\right]+\left[\nabla\times\left(\mathbf{H}^*\times\mathbf{H}\right)\right]\cdot\left[\left(\mathbf{H}^*\cdot\nabla\right)\mathbf{H}\right]\end{aligned} \tag{S6}$$

In order to make it concise, we only expand the first two terms on the right side of the equation (S6). The second two terms can be expanded in a similar way. Using vector algebra, we can write:

$$\nabla\times\left(\mathbf{E}^*\times\mathbf{E}\right) = \mathbf{E}^*\left(\nabla\cdot\mathbf{E}\right)-\mathbf{E}\left(\nabla\cdot\mathbf{E}^*\right)+\left(\mathbf{E}\cdot\nabla\right)\mathbf{E}^*-\left(\mathbf{E}^*\cdot\nabla\right)\mathbf{E} \tag{S7}$$

For a source-free medium, the first two terms of equation (S7) are zero and we have:

$$\begin{aligned}&\left[\nabla\times\left(\mathbf{E}^*\times\mathbf{E}\right)\right]\cdot\left[\left(\mathbf{E}^*\cdot\nabla\right)\mathbf{E}\right]=\left[\left(\mathbf{E}\cdot\nabla\right)\mathbf{E}^*-\left(\mathbf{E}^*\cdot\nabla\right)\mathbf{E}\right]\cdot\left(\mathbf{E}^*\cdot\nabla\right)\mathbf{E}\\&=\left[\left(\mathbf{E}\cdot\nabla\right)\mathbf{E}^*\cdot\left(\mathbf{E}^*\cdot\nabla\right)\mathbf{E}\right]-\left[\left(\mathbf{E}^*\cdot\nabla\right)\mathbf{E}\cdot\left(\mathbf{E}^*\cdot\nabla\right)\mathbf{E}\right]\\&=\left[\left(\mathbf{E}\cdot\nabla\right)\left(\mathbf{E}^*\cdot\nabla\right)\left(\mathbf{E}^*\cdot\mathbf{E}\right)\right]-\left|\left(\mathbf{E}^*\cdot\nabla\right)\mathbf{E}\right|^2\end{aligned}\tag{S8}$$

$$\begin{aligned}&\left[\nabla\times\left(\mathbf{E}^*\times\mathbf{E}\right)\right]\cdot\left[\left(\mathbf{H}^*\cdot\nabla\right)\mathbf{H}\right]=\left[\left(\mathbf{E}\cdot\nabla\right)\mathbf{E}^*-\left(\mathbf{E}^*\cdot\nabla\right)\mathbf{E}\right]\cdot\left(\mathbf{H}^*\cdot\nabla\right)\mathbf{H}\\&=\left[\left(\mathbf{E}\cdot\nabla\right)\mathbf{E}^*\cdot\left(\mathbf{H}^*\cdot\nabla\right)\mathbf{H}\right]-\left[\left(\mathbf{E}^*\cdot\nabla\right)\mathbf{E}\cdot\left(\mathbf{H}^*\cdot\nabla\right)\mathbf{H}\right]\\&=\left[\left(\mathbf{E}\cdot\nabla\right)\left(\mathbf{H}^*\cdot\nabla\right)\left(\mathbf{E}^*\cdot\mathbf{H}\right)\right]-\left[\left(\mathbf{E}^*\cdot\nabla\right)\left(\mathbf{H}^*\cdot\nabla\right)\left(\mathbf{E}\cdot\mathbf{H}\right)\right]\end{aligned}\tag{S9}$$

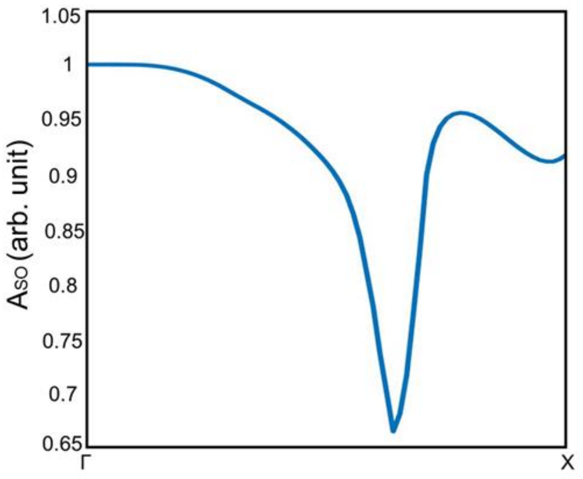

Figure S3: Normalized spin-orbit parameter A_{SO} as a function of wavevector *k*, calculated along Γ-X direction in the reciprocal space. All A_{SO} values are normalized to the value of A_{SO} at the Γ point (1.9 × 10^3).

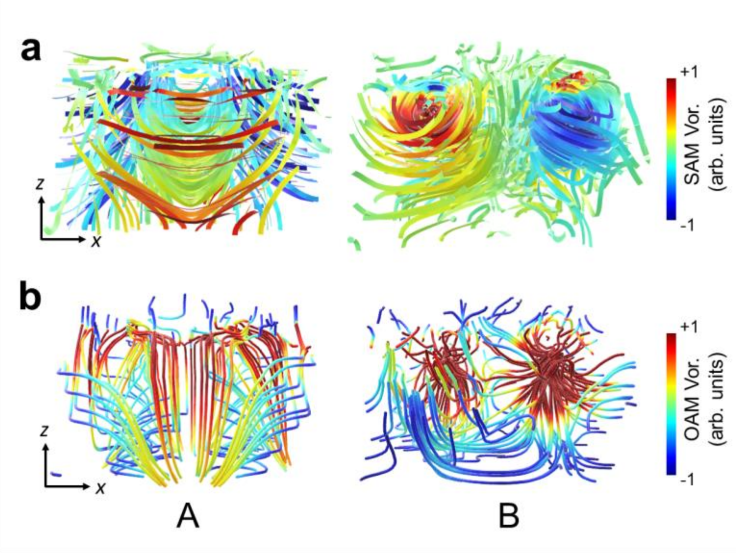

Figure S4: (a) Spin and (b) orbital angular momentum vorticities shown in *x*-*z* plane calculated at points A (left column) and B (right column) indicated in the band diagram of Figure 4a.

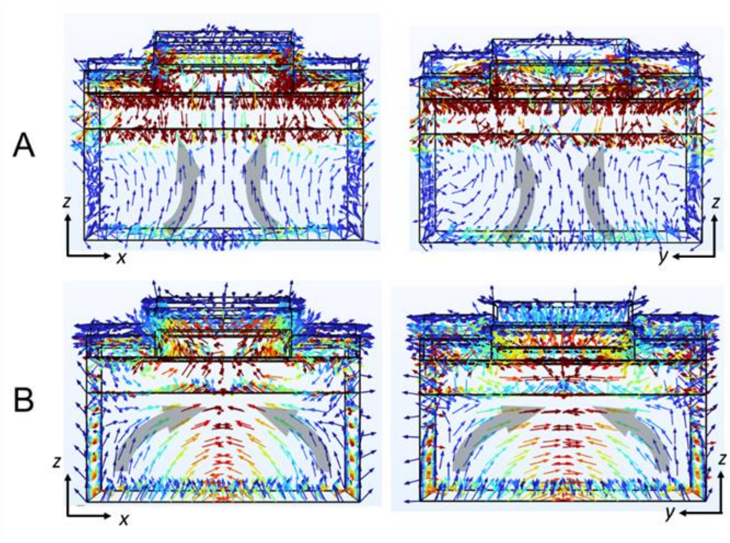

Figure S5: Total angular momentum vorticity in *x-z* (left) and *y-z* (right) planes calculated at points A (top) and B (bottom) indicated in the band diagram of Figure 4a. The arrows show the total angular momenta calculated for bands A and B flow in opposite directions.

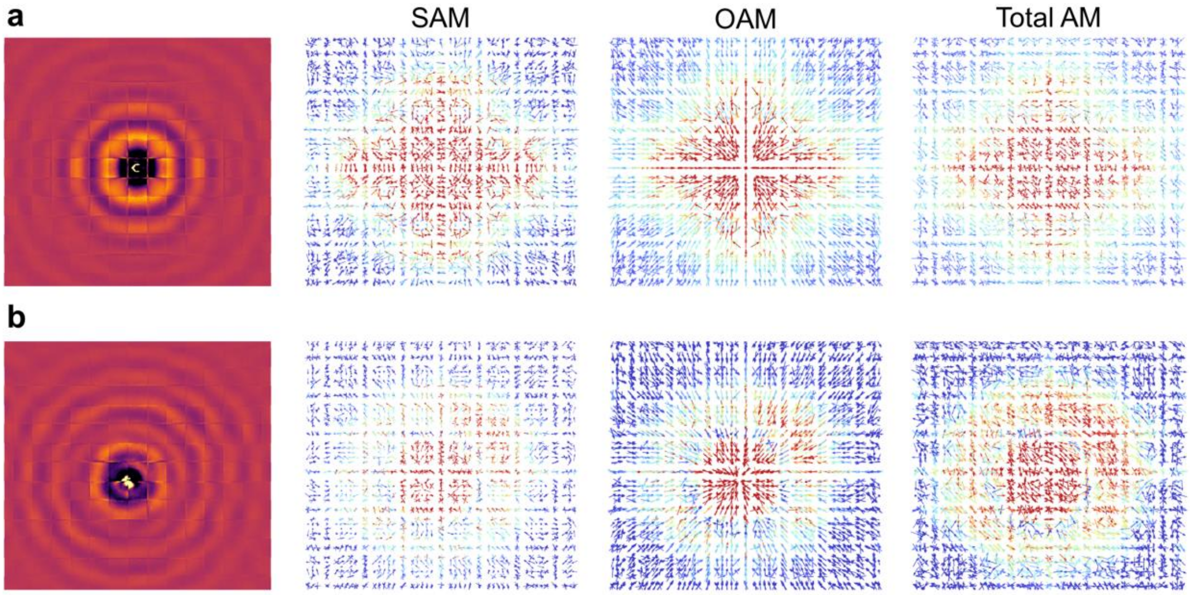

Figure S6: Electric field, SAM, OAM and total AM in *x-y* plane when the sample is excited by an electron beam (a) at the site where the ITO thickness is h_1= 65 nm and (b) at the crossing point of the four sectors with different ITO heights, as indicated in Figure 3c and 3e, respectively.

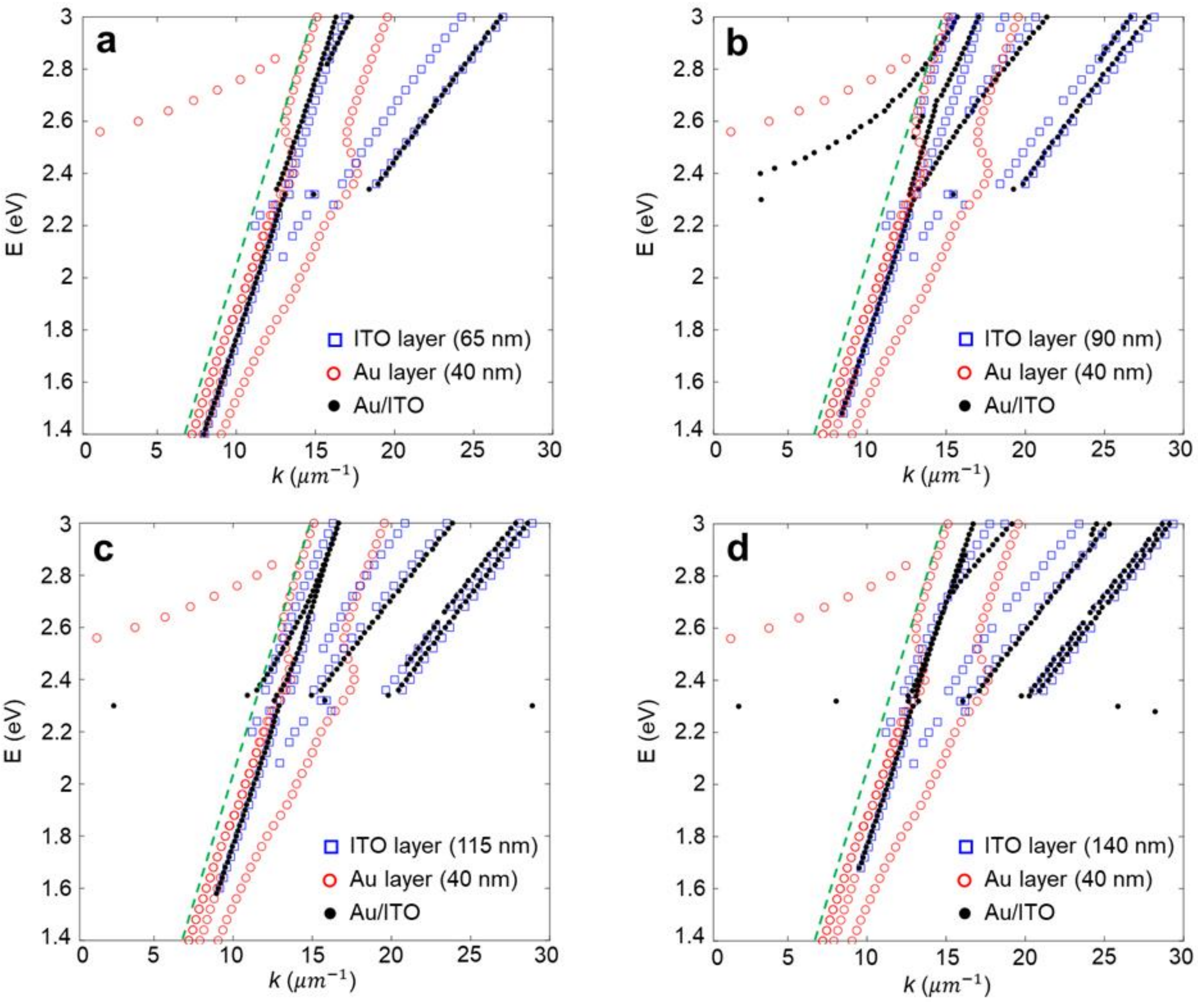

Figure S7: Dispersion diagram of ITO layers (blue squares) with different thicknesses: (a) 65 nm, (b) 90 nm, (c) 115 nm, (d) 140 nm, 40-nm-thick Au layer (red circles), and Au-coated ITO layer (black circles). The light line is indicated by a green dashed line. No hybridization of photonic and/or plasmonic modes occurs in our energy range of interest wherein, according to Figure 3a, the SOC phenomena occurs (E < 2 eV).

References

[1] J. Buhl, D. Yoo, M. Köpke, M. Gerken, "Two-Dimensional Nanograting Fabrication by Multistep Nanoimprint Lithography and Ion Beam Etching," *Nanomanufacturing*, vol. 1, pp. 39–48, 2021. https://doi.org/10.3390/NANOMANUFACTURING1010004.

[2] K. Y. Bliokh, A. Y. Bekshaev, F. Nori, "Extraordinary momentum and spin in evanescent waves," *Nat. Commun.,* vol. 5, pp. 1–8, 2014. https://doi.org/10.1038/ncomms4300.

[3] C. Taneja, D. Paul, G. V. Pavan Kumar, "Experimental observation of transverse spin of plasmon polaritons in a single crystalline silver nanowire," *Appl. Phys. Lett.*, vol. 119, pp. 161108, 2021. https://doi.org/10.1063/5.0055788.
